# ARV-825 Showed Antitumor Activity against BRD4-NUT Fusion Protein by Targeting the BRD4

**DOI:** 10.1155/2023/9904143

**Published:** 2023-12-14

**Authors:** Liu Yang, Yue Jing, Xia Xia, Xiushan Yin

**Affiliations:** ^1^Applied Biology Laboratory, College of Pharmaceutical and Biological Engineering, Shenyang University of Chemical Technology, Shenyang 110142, China; ^2^State Key Laboratory of Proteomics, Beijing Proteome Research Center, National Center for Protein Sciences (Beijing), Beijing Institute of Lifeomics, Beijing 102206, China; ^3^Roc Rock Biotechnology (Shenzhen), Shenzhen 518118, China

## Abstract

**Objective:**

The bromodomain-containing 4 (BRD4) is a member of the bromodomain and extra terminal domain (BET) family, which is an important epigenetic reader. It is currently a promising oncology target. In some tumors, BET bromodomain inhibitors have demonstrated promising results. Proteolysis-targeting methods (PROTAC), which rapidly and effectively degrade BRD4, have displayed considerable potential in the treatment of tumors in recent years. The purpose of this study is to examine the potential impact of BRD4 PROTAC compounds ARV-825 on oncogene BRD4-NUT fused protein in NUT carcinoma.

**Methods:**

The effectiveness of ARV-825 was evaluated at the cellular level using the cell counting kit 8 test, wound healing, cell transfection, western blotting analysis, and RNA sequencing. The effectiveness of ARV-825 was also examined in vivo using a xenograft model.

**Results:**

The BRD4-NUT fusion gene was overexpressed in 3T3 cells, and the pathogenic fusion gene was simulated. The results showed that the overexpression of BRD4-NUT could promote the proliferation and migration of 3T3 cells, but the expression of BRD4 protein was degraded after the addition of the novel cereblon-based PROTAC compound ARV-825 against BRD4, resulting in inhibition of BRD4-NUT 3T3 cell proliferation and migration. Further RNA-seq analysis showed that overexpression of BRD4-NUT was accompanied by increased expression of gene (e.g., Myc, E2F, TRAFs, Wnt, Gadd45g, and Sox6) with significantly enriched pathway (e.g., small cell lung cancer, NF-kappa B signaling pathway, and breast cancer), promoted cell cycle from G 1 phase to S phase, and increased cell proliferation and migration, activated the antiapoptosisi signal, led to abnormal cell growth, and ultimately led to tumorigenesis. The addition of ARV-825 effectively rescued this process and effectively inhibited cell vitality, proliferation, and migration. In vivo studies demonstrated that treatment with ARV-825 greatly suppressed tumor growth without causing harmful side effects and downregulated the BRD4-NUT expression level.

**Conclusion:**

Through the induction of BRD4 protein degradation, ARV-825 can successfully limit BRD4-NUT 3T3 cell proliferation in vitro and in vivo. These findings suggested that the BRD4 inhibitor ARV-825 would be an effective therapeutic strategy for treating NUT carcinoma that with the genetic feature of BRD4-NUT fusion event.

## 1. Introduction

NUTM1 (also known as NUT) gene rearrangement could lead to NUT carcinoma (NC), which is an aggressive subtype of squamous cell carcinoma. NC primarily acts on body midline organs. It presents as a monomorphic low differentiated squamous cell carcinoma [1]. Chromosomal translocation caused by BRD4-NUT fusion is the most common because of the genetically characterized disease, NC. The age of onset of this cancer ranges from 3 to 78 years [1]. NC is nearly fatal and is almost entirely resistant to currently known treatments, and even with aggressive chemotherapy, the typical survival period following diagnosis with NC is less than one year (9.5 months) [1].

The primary member of the bromodomain and extra terminal domain (BET) family of proteins is the bromodomain-containing protein 4 (BRD4), which has two bromodomains (BD) and one extra terminal (ET) domain [2–5]. BDs are capable of recognizing and interacting with lysine in acetylated histones [6]. The ET domain attracts transcriptional regulators, boosting the expression of several important oncogenes, such as c-myc, bcl-xl, and cyclin d1 [7]. BRD4 dysfunction is associated with the tumorigenesis and progression of a variety of cancers, including acute myeloid leukemia, colon cancer, Burkitt's lymphoma, breast cancer, diffused large B-cell lymphoma, multiple myeloma, and ovarian cancer [4, 8–12]. Additionally, it is the site of a chromosomal translocation between chromosomes 15 and 19, which is the cause of aggressive NUT carcinoma that normally manifests as a single *t* (15; 19) translocation and gives rise to the novel fusion oncogene BRD4-NUT [7].

3T3 cells, a type of mouse embryonic fibroblast cell line, have become one of the most widely studied cell lines in biology since their isolation by George Todaro and Howard Green in the 1960s [13]. These cells are commonly used in research for a variety of applications, including cell culture, transfection studies, and tumorigenicity assays. Morphologically, 3T3 cells are small and spindle-shaped with elongated nuclei, growing as adherent monolayers in tissue culture. Their ability to undergo contact inhibition, which means they stop dividing when they come into contact with other cells, is a distinguishing feature [14]. Despite their nontumorigenic nature, 3T3 cells are used as a model system for studying cellular transformation and cancer progression. Introducing oncogenes or chemical carcinogens can transform these cells into cancer cells. The use of 3T3 cells has provided valuable insights into the molecular mechanisms underlying cancer development [15]. In our study, we introduced the BRD4-NUT oncogene into 3T3 cells to facilitate the formation of tumors both in vitro and in vivo, thereby simulating nut carcinoma and enabling further investigation.

A number of BET inhibitors (BETis) have been developed during the past ten years and put through clinical studies to ascertain their potent antitumor effects for the treatment of human cancer [16]. The effectiveness of JQ1 was assessed in a variety of tumor types, and it could target superenhancers found in tumor-related genes and demonstrate antiproliferative and proapoptotic activity in a number of cancers [16, 17], including pancreatic ductal adenocarcinoma [18], renal cell carcinoma [19], breast cancer [20], medulloblastoma [21], and gastric cancer [22, 23]. As an illustration, the JQ1 analog chemical OTX015, a BRD4 inhibitor, is now undergoing clinical phase I studies to treat patients with solid tumors and hematologic malignancies [17, 20, 22, 23].

Although earlier research suggested that BETi like JQ1 and OTX015 have the potential to prevent tumor growth, they are not without drawbacks. Due to JQ1 and OTX015 inhibiting reversibility, BRD4 protein reaccumulates and MYC is only partially suppressed [24], necessitating the use of higher inhibitor doses. In contrast, inhibitors using proteolysis-targeting chimera (PROTAC) technology were developed to suppress BRD4 with better efficiency [25].

The PROTAC molecule is a bifunctional protein hydrolysate consisting of a ligand of the protein of interest (POI) and a covalently linked ligand of the E3 ubiquitin ligase (E3). Upon binding to POI, PROTAC can recruit E3, causing POI to ubiquitinate and then undergo proteasome-mediated degradation. It has been demonstrated that ARV-825, which combines a BRD4 inhibitor with a cereblon ligand using PROTAC technology, showed to be more effectively breaking down BRD4, inhibiting tumor growth effectively and consistently [12]. The antitumor effect of ARV-825 was systematically studied in pancreatic cancer [26, 27], melanoma resistant to vemurafenib [28], cholangiocarcinoma [29], thyroid carcinoma [30], acute myeloid leukemia [31, 32], T-cell acute lymphoblastic leukemia, and neuroblastoma [33, 34]. ARV-825 has not been clinically used in the targeted treatment of NUT carcinoma. Here, we took the advantage of ARV-825 to target BRD4-NUT and tested the antitumor activity in vitro and in vivo by using a BRD4-NUT fusing construct in 3T3 cells. The purpose of this study is to confirm ARV-825 anticancer effect against BET proteins in NUT carcinoma as well as potential underlying mechanisms.

## 2. Materials and Methods

### 2.1. Cell Line

3T3 and 293T-cell lines were preserved by the Applied Biology Laboratory of Shenyang University of Chemical Technology. Cells were sustained by providing 5% CO_2_ at 37°C, cultivated in the DMEM (Dulbecco's Modified Eagle's Media) medium (Thermo Fisher Scientific), medium with 100 U/mL penicillin-streptomycin (Millipore Sigma), and 10% fetal bovine serum (FBS) (ExCell Bio).

### 2.2. Plasmid

The pLV-EGFP T2A Puro-EF1A hNUTM1 plasmid was synthesized from VectorBuilder; the pcDNA3.1-CMV-BRD4-flag plasmid was purchased from YouBio. For the construction of pcDNA3.1-CMV-BRD4 NUT plasmid, the NUT target gene was amplified by using PCR, the forward 5′-aaacaggtcctgccctcgagGTTACTCTGGGTCCTGGACCTG-3′ and the reverse 5′-gggccctctagactcgagCTGGCTACGACGTCGTTTCTTC-3′ primers were applied. PCR conditions: 95°C for 2 minutes, then 35 cycles of 95°C for 30 seconds, 50°C for 30 seconds, and 72°C for 4 minutes, followed with an extention cycle of 72°C for 7 minutes. pcDNA3.1-CMV-BRD4–FLAG plasmid was digested with XhoI. Then, the plasmid DNA was isolated from the transformed DH5*α* cells and individual bacterial colonies, ligated in the digested product (EasyGeno kit), and sequenced using the aforementioned primers.

### 2.3. Cell Transfection

The 3T3 cells were electroporated with pcDNA3.1-CMV-BRD4 NUT (3 *μ*g). Cells were gently resuspended in 1.5 mL of prewarmed DMEM media following transfection. All cells were cultivated at 37°C in the incubator with 5% CO_2_. The following day, the media was changed to complete the DMEM medium, and cells were kept as before. After 48 h, the geneticin (Sigma-Aldrich) was applied to select for stable cell lines.

### 2.4. Western Blotting

3T3 cells and BRD4-NUT 3T3 cells were cultured in 12-well plates. BRD4-NUT 3T3 cells were treated with 0.003 *μ*M and 0.03 *μ*M ARV-825 for 72 h. Then, whole cells were lysed in a lysis buffer. Proteins were determined by using the Pierce bicinchoninic acid (BCA) protein assay kit (#PA115, TIANGEN, China). The 10% SDS-PAGE gels used to separate the whole protein samples (20 *µ*g proteins per treatment in each lane) were then transferred to a polyvinylidene fluoride (PVDF) membranes (#FFP39, Beyotime, China). The membrane was sealed with 5% skim milk to bind to nonspecific cells, after blocking incubated with primary antibody at 4°C overnight. The primary antibodies include BRD4 (#E2A7X, Cell Signaling Technology, USA), NUT (#C52B1, Cell Signaling Technology, USA), and *β*-actin (#8H10D10, Cell Signaling Technology, USA). Then, followed by incubation at room temperature for 1 h with the appropriate HRP-labelled secondary antibody (1 : 1000 dilution), an ECL kit (#P0018AS, Beyotime, China) was used to identify the antibody and antigen binding.

### 2.5. Cell Counting Kit-8 Assay

Plate the cells to be tested in a 96-well plate, 5000 cells/well. Treat the cells according to the experimental design. Add the CCK-8 reagent to the cells, ensuring that each well is covered with sufficient volume. Incubate the cells for a period of 1–4 h at 37°C in the presence of CO_2_. Measure the absorbance of the CCK-8 solution at 450 nm using a microplate reader. The data can be analyzed using various statistical methods to determine the effects of the experimental treatment on cell proliferation. Additional controls, such as a blank well and a positive control well, are often included to ensure the accuracy of the assay.

### 2.6. Wound Healing Assay

Cells are first plated in a cell culture dish and grown until they reach 100% confluency, forming a single layer of cells. A linear wound is then created on this monolayer using a sterile tool. The dish is then washed to remove any loose cells with PBS and add fresh DMEM media containing the ARV-825 compounds. The wound is monitored every 12 h using a microscope, and images are taken at regular intervals to track the movement of cells into the wound. The speed and direction of cell migration can be quantified using ImageJ software. Area recovery rate = (initial wound width—remaining wound width)/initial wound width × 100%.

### 2.7. RNA-Sequencing and Analysis

3T3 cells and BRD4-NUT 3T3 cells were cultured in 6 cm discs. BRD4-NUT 3T3 cells were treated with 0.003 *μ*M and 0.03 *μ*M ARV-825 for 48 h. After that, cells were collected and RNA molecules were extracted from cells using Trizol kits from Invitrogen. The quality and quantity of RNA are then assessed using NanoDrop. In library construction, RNA is converted into cDNA using reverse transcription with random hexamers. The resulting cDNA fragments are then fragmented, and adapters containing indexes are added to each fragment. This step enables multiplexed sequencing, and the resulting libraries are amplified using PCR. All purified libraries were sequenced on DNBSEQ-T7RS of Geneplus to acquire 150 bp paired-end sequence reads. The raw sequencing data undergo quality control preprocessing steps and removal of ribosomal RNA sequences before the processed reads are aligned to a reference genome or transcriptome using software STAR. Finally, the expression levels of genes are quantified using feature counts, and differential expression analysis can be performed using statistical methods edgeR.

### 2.8. Quantitative Real-Time PCR (RT-qPCR)

3T3 cells and BRD4-NUT 3T3 cells were cultured in 6-well discs. The BRD4-NUT 3T3 cells were then treated with 0.003 *μ*M and 0.03 *μ*M ARV-825 for 48 h. Following this, total RNA was extracted from BRD4-NUT 3T3 cells using Trizol (Vazyme) in accordance with the manufacturer protocol. Real-time quantitative PCR was employed to detect mRNA using the Quant Studio TM Design and Analysis Software. To synthesize cDNA, the Prime-Script RT reagent kit (TaKaRa Biotech) was utilized. Real-time PCR using SYBR Premix Ex Taq (TaKaRa Biotech) was used to measure the mRNA levels of genes, including Sox6, Cnn1, Traf1, and Eid3. These levels were calculated as a ratio to the endogenous Gapdh mRNA level. Amplification followed a protocol of denaturation at 95°C for 1 min, followed by 40 cycles of 95°C for 15 s, 60°C for 15 s, and 72°C for 45 s. Each primer was designed to span an exon-exon junction, and the generation of individual amplicons was examined in melting curve assays. Lastly, the expression levels of differentiation genes were analyzed using the 2^−ΔΔCT^ method.

### 2.9. In Vivo Xenografts

The Institutional Animal Care and Use Committee (IACUC) of the authors' institutions gave its approval for the ethical care of the animals used in the research. We bought nude mice from Lan puda, Ltd. (Shenyang, China). Eight million 3T3-BRD4-NUT cells were subcutaneously implanted into the backs of four-week-old male nude mice (*n* = 6 per group), and when the engrafted tumor reached a size of around 200 mm^3^, either 10 mg/kg of ARV-825 or the vehicle control was administrated intraperitoneally each day. When the tumor size in the control group reached 1,000 mm^3^, the mice were sacrificed. Every three days, the size of the subcutaneous tumor was measured using calipers. Tumor volume was determined using the formula (width × width × length × 0.52).

## 3. Results

### 3.1. Overexpression of BRD4-NUT Promotes Cell Proliferation and Migration in 3T3 Cells

**Figure 1:**
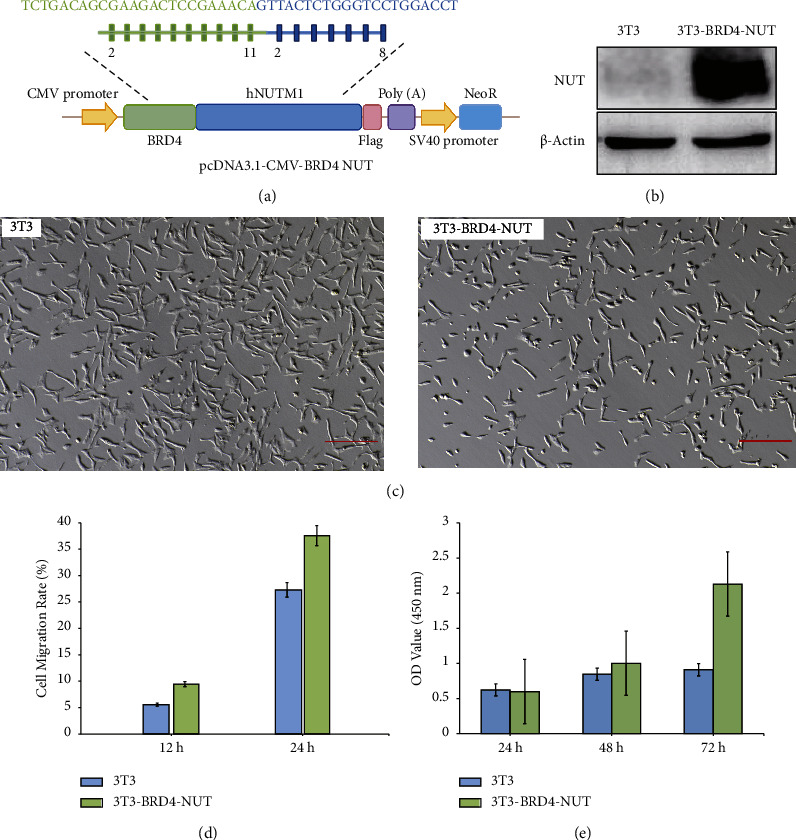
Overexpression of BRD4-NUT promotes cell proliferation and migration. (a) CMV-BRD4-NUT vector construction scheme. (b) 48 h after transfection of 3T3 cells with BRD4-NUT vectors, western blot analysis was conducted. (c) Bright field of 3T3 cells, 3T3-BRD4-NUT cells. Scale bars, 200 *μ*m. Cells were further cultured in a complete medium for indicated time periods. (d) Cell migration (wound healing assays). (e) Cell proliferation (CCK8 OD 450 nm) was tested. Data were presented as the mean ± standard deviation (SD, *n* = 3) (same for all Figures). The experiments were repeated three times, with similar results obtained.

Given that the BRD4-NUT fusion protein is the oncoprotein for NUT carcinoma [35], we constructed CMV-BRD4-NUT plasmid which can overexpress BRD4-NUT to simulate the pathogenic fusion gene of NUT carcinoma (Figure 1(a)). Exons 2–11 of BRD4 and 2–8 of hNUTM1 were seamlessly connected, mimicking the BRD4-NUT fusion in NC patients [36]. Western blotting showed that BRD4-NUT was overexpressed in 3T3 cells (Figure 1(b)), but there was no appreciable morphology difference between 3T3-BRD4-NUT and 3T3 cells (Figure 1(c)). After overexpression of BRD4-NUT fusion gene, migration ability of 3T3 cells was significantly enhanced (Figure 1(d)). The CCK8 assay found there was an abnormal increase in cell proliferation demonstrated that BRD4-NUT overexpression could significantly promote 3T3-BRD4-NUT cell proliferation (Figure 1(e)), which could be attributed to the loss of cell contact inhibition. In conclusion, these results showed that we successfully constructed a stable cell line that overexpressed BRD4-NUT fusion protein and found that overexpression of BRD4-NUT could promote the proliferation and migration of 3T3 cells.

### 3.2. ARV-825 Inhibits the Proliferation and Migration of 3T3-BRD4-NUT Cells

**Figure 2:**
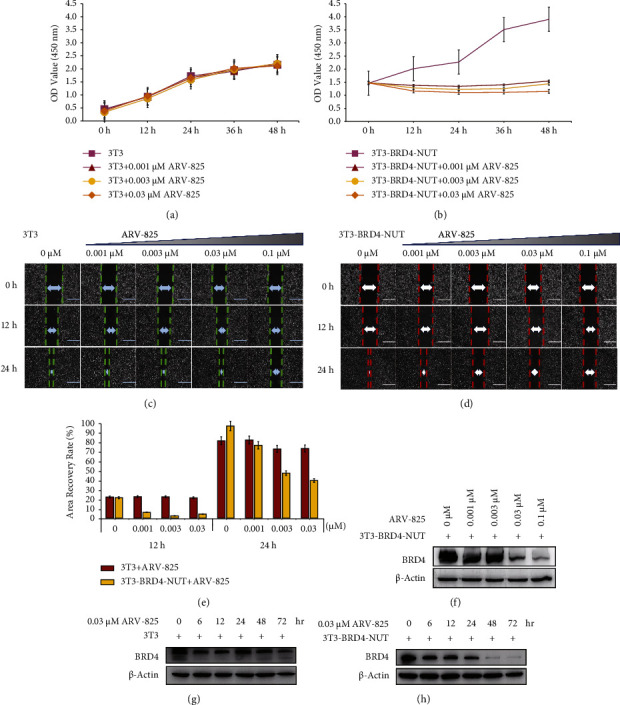
ARV-825 suppresses the proliferation and migration of BRD4-NUT 3T3 cells. (a) Different concentrations of ARV-825 had little effect on the proliferation of 3T3 cells. (b) Cells overexpressing BRD4-NUT decreased cell proliferation after being treated with ARV-825 at different concentrations in 3T3 cells. (c) 3T3 cell and (d) 3T3-BRD4-NUT cell migration changes after treatment with different concentrations of ARV-825 at specific times. Scale bars, 200 *μ*m. (e) Cells overexpressing BRD4-NUT decreased cell wound recovery after being treated with ARV-825 in 3T3 cells. (f) Western blotting analysis showing the BRD4 protein level in 3T3-BRD4-NUT cells after being treated with ARV-825 at different concentrations for 72 h. (g) 3T3 cells and (h) BRD4-NUT 3T3 cells were treated with 0.03 *μ*m ARV-825 for different times, and the changes of BRD4 protein expression were detected with western blotting.

In order to assess the toxic effect of ARV-825, wild-type 3T3 cells were cultured in varying concentrations of ARV-825 (0.001–0.03 *µ*M), and it was found that there was almost no toxic effect on wild-type 3T3 cells (Figure 2(a)). Meanwhile, for the overexpressing BRD4-NUT cell lines, cells were treated with various doses of ARV-825 (0–0.1 *µ*M) for 48 hours to assess the drug effect on the cells. After receiving ARV-825 treatment, 3T3-BRD4-NUT cell proliferation was decreased in a dose-dependent manner, and ARV-825 showed the inhibition effect even at the lowest concentration tested 0.001 *µ*M (Figure 2(b)). Wound healing assay results showed that ARV-825 (0.001–0.03 *µ*M, 24 h) treatment failed to suppress 3T3 cell migration and proliferation, but ARV-825 at 0.1 *μ*M was suppressed to 3T3 cell migration and indicated that 0.1 *µ*M ARV-825 is toxic to 3T3 cells (Figures 2(c) and 2(d)). ARV-825 (0.001–0.03 *µ*M, 24 h) significantly reduced the number of migrating 3T3-BRD4-NUT cells. Again, ARV-825 at 0.1 *µ*M significantly suppressed the migration of 3T3-BRD4-NUT cells, demonstrating a dose-dependent effect (Figure 2(e)). These findings showed that ARV-825 demonstrated negligible toxicity towards 3T3 wild-type cells, and it was able to effectively reduce the proliferation and migration of 3T3-BRD4-NUT cells. After 72 h of continuous ARV-825 treatment at different concentrations, western blotting analysis showed that ARV-825 dose-dependently suppressed the BRD4-NUT protein level in 3T3-BRD4-NUT cells (Figure 2(f)). Meanwhile, we observed that when 0.03 *μ*m ARV-825 was treated to BRD4-NUT 3T3 cells at different times, the drug began to take effect from 24 hours, resulting in a reduction of the expression level of the BRD4-NUT fusion protein (Figures 2(g) and 2(h)).

### 3.3. Comparative Analysis of Gene Transcript Abundance

**Figure 3:**
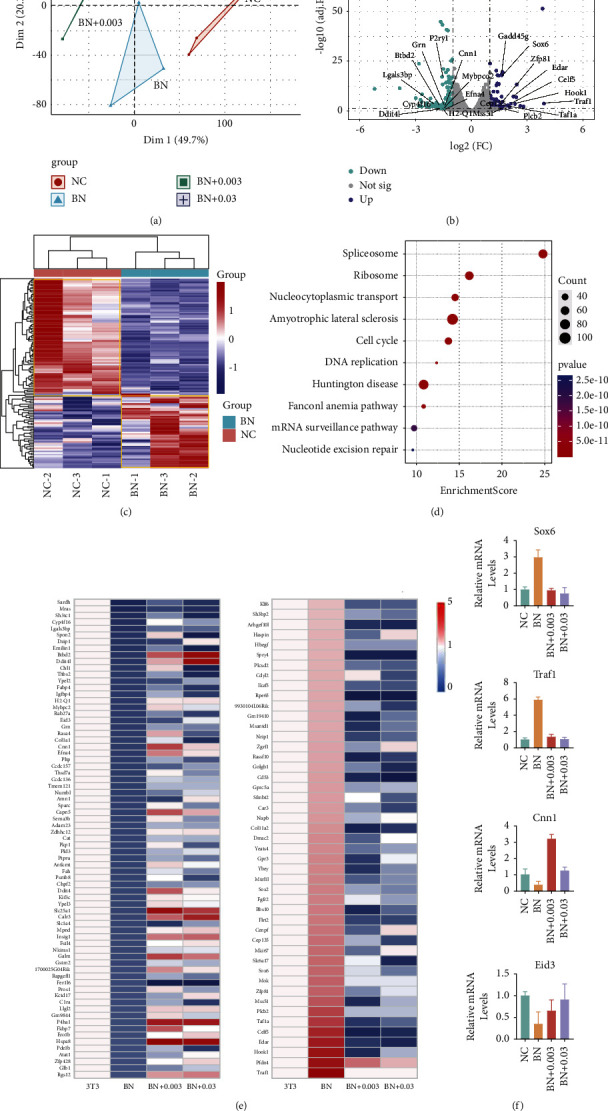
Transcriptome difference revealed dynamic changes with ARV-825 treatment. (a) PCA analysis of four sets of data. A projection of the input data set onto the first two principal components. BN, 3T3-BRD4-NUT cells, BN + 0.003, 0.003 *μ*M ARV-825 treated with 3T3-BRD4-NUT cells, BN + 0.03, 0.03 *μ*M ARV-825 treated with 3T3-BRD4-NUT cells. (b) Volcano plot of RNA-seq analysis of gene expression changes in BRD4-NUT 3T3 and control cells. Genes highlighted in blue form the up-fold changes, and cyan indicates down regulated genes and black on behalf of unchanging genes. (c) Heatmap view of the differentially expressed genes in 3T3-BRD4-NUT cells compared with 3T3 cells. Red indicates upregulation, and blue indicates downregulation. (d) KEGG pathway enrichment analysis significantly upregulated after BRD4-NUT overexpression compared with 3T3 cells (top 10). The color represents significance, and the circle size represents the number of genes enriched. (e) The top differentially expressed genes that before and after ARV-825 treatment were subjected to functional enrichment analysis cells (*p* < 0.05, |log2FC| > 1). Each column represents a different sample, and each row represents a single gene. Color changes within a row indicate expression levels relative to the average of the same population. Red indicates upregulation, blue indicates downregulation, and white indicates the basic level of expression. (f) q-PCR analysis relative gene expression levels of Sox6, Traf1, Cnn1, and Eid3 in each group. Data were presented as the mean ± SD. (*n* = 4).

**Figure 4:**
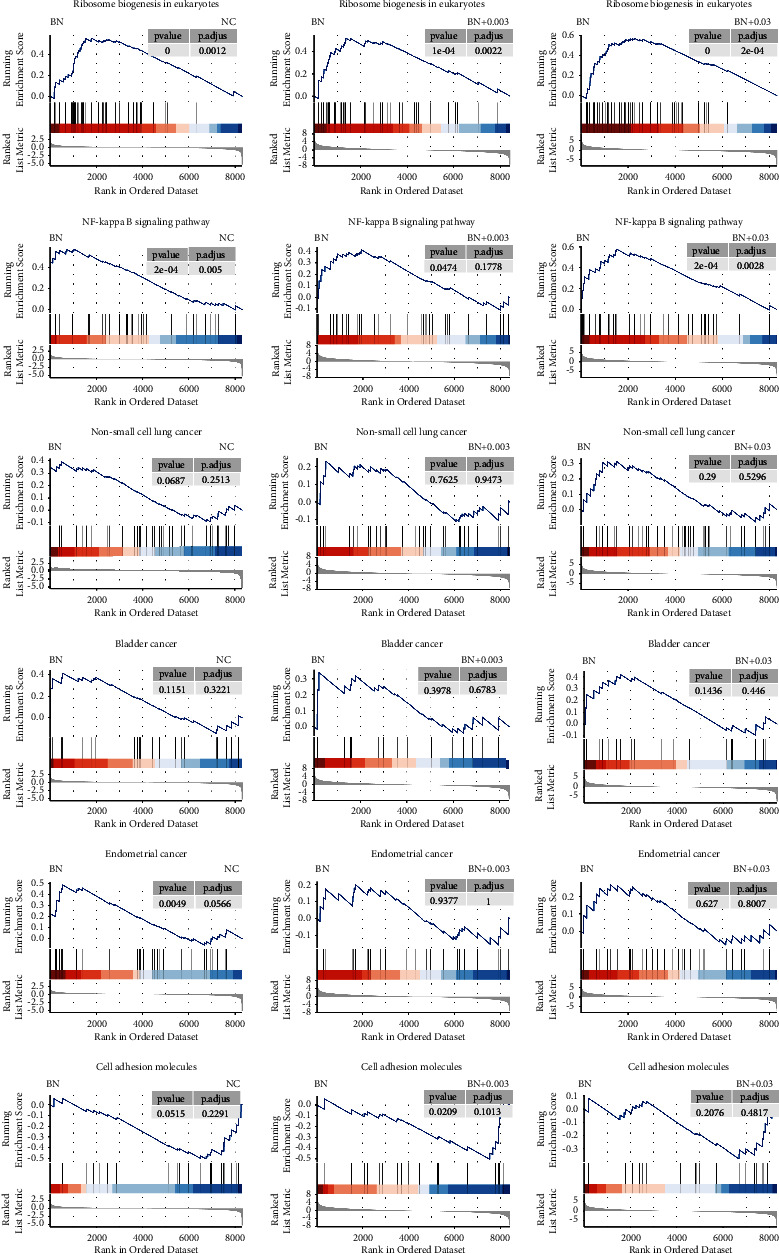
GSEA plots showing the enrichment of genes in the gene set in RNA-Seq following ARV-825 treatment.

Next, we performed RNA-seq to investigate the potential mechanism of BRD4-NUT at the transcriptional level. We tested four sets of data consisting 3T3 cells, 3T3-BRD4-NUT cells, 0.003 *µ*M, and 0.03 *µ*M ARV-825-treated 3T3-BRD4-NUT cells. PCA analysis separated the four groups. The low concentration of 0.003 *µ*M ARV-825 treatment clustered with 0.03 *µ*M ARV-825, not with BRD4-NUT, indicating the sensitive drug treatment (Figure 3(a)). According to Figure 3(b), when the transcriptome data from two paired 3T3 cells with pre- and post-overexpressing BRD4-NUT were compared under the conditions of |log2fold change| > 1 and an adjusted *p* < 0.01, 103 genes were found to be upregulated, and 159 genes to be downregulated. Those genes showed significantly different expression levels in 3T3-BRD4-NUT cells compared with 3T3 cells. Correlation analysis of the dysregulated genes was performed and visualized in a heat map (Figure 3(c)). We found that genes involved in spliceosome, ribosome, nucleocytoplasmic transport, amyotrophic lateral sclerosis, cell cycle, and Fanconi anemia pathway were significantly upregulated, indicating that these pathways may associate with overexpression of BRD4-NUT (Figure 3(d)). However, after the addition of ARV-825, the abnormally up-down-regulated genes caused by overexpression of BRD4 changed, showing the rescue trend at the transcriptional level (Figure 3(e)). We conducted an experiment to examine the expressions of Sox6, Traf1, cnn1, and Eid3 genes. Upon overexpression of BRD4-NUT, we observed a significant upregulation in the expressions of Sox6 and Traf1genes, while there was a significant downregulation in the expressions of Cnn1 and Eid3 genes. Subsequently, upon application of ARV-825 to BRD4-NUT 3T3 cells, we noted a downregulation in the expressions of Sox6 and Traf1 genes, along with an upregulation in the expressions of Cnn1 and Eid3 genes. Overtime, the expressions gradually returned to the levels observed in wild-type 3T3 cells, indicating a rescue trend that was in line with the data obtained from RNA-seq (Figure 3(f)). Similarly, in the GSEA enriched pathway, after the addition of ARV-825, the pathway changes also showed a rescue trend (Figure 4).

### 3.4. ARV-825 Suppresses Tumor Growth in the Xenograft Tumor Model

**Figure 5:**
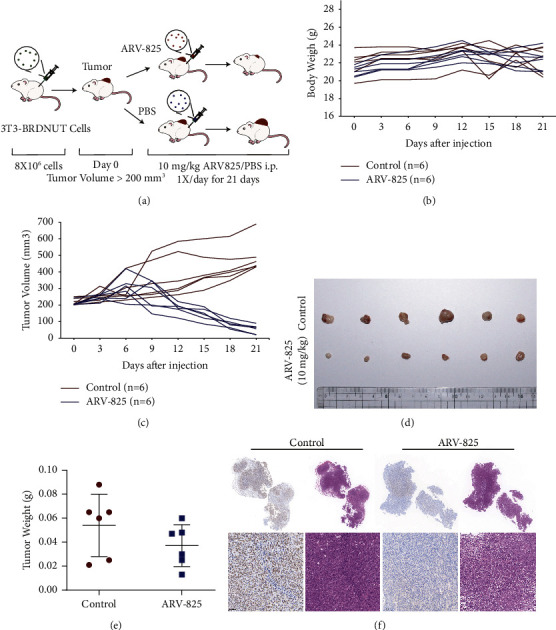
ARV-825 displays antitumor efficacy in the BRD4-NUT 3T3 xenograft model. (a) Diagram of xenograft flowchart, nude mice bearing xenograft tumors were treated by either 10 mg/kg ARV-825 or vehicle control intraperitoneally every day for 21 days. Data are the mean ± SEM (*n* = 6). (b) Mice body mass was weighed every 3 days. (c) Tumor volume was recorded every 3 days and calculated using the formula: (width × width × length × 0.52). (d) Photograph of xenograft tumors from ARV-825-or vehicle-treated mice. (e) Tumor size at the endpoint. (f) Histological characteristics of subcutaneous tumors (H&E staining) and immunohistochemical staining of NUTM1 protein (representing brd4-NUTM1 fusion protein) in mice treated with ARV-825 and PBS control.

Using 3T3-BRD4-NUT cells, a xenograft model of NUT carcinoma cancer was established in order to examine the anticancer effect of ARV-825 in vivo (Figures 5(a)). When the volume of the subcutaneous tumor reached approximately 200 mm^3^, nude mice received daily intraperitoneal injections of ARV-825 at a dose of 10 mg/kg. In comparison to the control group, the tumor burden in the ARV-825 therapy group was significantly decreased (Figures 5(b)–5(d)). We observed that treatment with ARV-825 caused a reduction in tumor weight compared to the PBS-treated control (Figures 5(e)). As shown in Figure 5(f), NUTM1 labelling was markedly reduced in the ARV-825-treated group compared to the PBS control group, indicating the potential efficacy of ARV-825. In addition, the body weights did not significantly differ from the treatment group and control group (Figure 5(b)). These findings showed that ARV-825 might significantly slow the growth of NUT carcinoma tumors without causing any evident negative effects.

## 4. Discussion

NUT carcinoma is a rare, aggressive, and lethal arising carcinoma. As previously mentioned, *t* (15; 19) translocation generated BRD4-NUT fusion oncogene that plays a key pathological role in midline carcinoma. Despite aggressive chemotherapy and radiotherapy, the typical survival for NC patients is less than 1 year (9.5 months). NC still lacks effective treatments and strategies. Finding therapeutic targets to treat NUT carcinoma and comprehending the process of its incidence and progression are urgent and crucial tasks.

In NUT carcinoma, which is frequently caused by the fusion of the bromodomains of BRD3 or BRD4, the oncogenic characteristics of BET proteins were initially identified [37]. BET proteins became promising therapeutic candidates for the treatment of inflammation and cancer with the invention of numerous small chemical inhibitors that were specifically bound to BET bromo domains, such as benzodiazepine derivatives, I-BETs, and JQ1 [38–41]. JQ1 replaces BET proteins from acetylated lysine on chromatin by competitively binding to the BET bromodomain [39]. In numerous distinct hematological malignancies, inhibiting the BET bromodomain with JQ1 has shown potent anticancer effects both in vitro and in vivo [9–11, 39, 42]. However, they also have drawbacks. Cell lines generated from solid tumors, such as those from cervical, breast, and lung malignancies, seem to be less resistant [11, 43]. Additionally, it has been demonstrated that BRD4 inhibitors may bind to BRD4 reversibly and inhibit it partially, significantly impairing anticancer efficacy [44].

Unlike BRD4 inhibitors, ARV-825 is a new chimeric molecule that Lu et al. have created to efficiently degrade BRD4 using the PROTAC platform [44]. ARV-825 had a more effective inhibitory effect on the BRD4 protein. BRD4 protein is intensely and persistently degraded as a result of ARV-825 directed recruitment of BRD4 into the E3-ubiquitin ligase cereblon [44]. ARV-825 caused more apoptosis induction and more efficient proliferation suppression, which may be due to its quick and long-lasting BRD4 degradation and inhibition of downstream targets such as c-myc [45]. Lu et al. found that ARV-825 can degrade BRD4 protein, thus showing effective effect on cholangiocarcinoma cells [29]. Consistent with the results, we found that treatment with ARV-825 led to decline in BRD4 levels in 3T3-BRD4-NUT cells.

The effects of ARV-825 on 3T3-BRD4-NUT cell gene expression were demonstrated by RNA-seq and western blotting analysis. The results showed that inhibition of BRD4 by ARV-825 resulted in changes in the mRNA of E2f, Trafs, Wnt, Gadd45g, and Sox6 in 3T3-BRD4-NUT cells. Further investigation of the NUT carcinoma RNA-seq data may identify novel therapeutic targets and precise signaling mechanisms.

ARV-825 inhibited the overexpression of BRD4-NUT tumor growth in the 3T3 cell xenograft model. In accordance to in vitro findings, ARV-825 could reduce BRD4 protein levels in vivo. This further demonstrated that ARV-825 may effectively regulate the critical BRD4-NUT gene regulation network. Additionally, it indicated that there were no statistically significant differences in body weight between mice getting ARV-825 treatment and the control group. Organs from mice treated with ARV-825 did not show any other noticeable negative effects. One study found that mice treated with JQ1 had decreased body weight and impaired adipogenesis, but no significant toxic effects were observed in ARV-825-treated organs in addition to body weight [46]. These findings make it quite evident that ARV-825 is both safe and effective. Our research has demonstrated that targeted degradation through ubiquitination is a promising treatment approach for NUT carcinoma. Currently, we are the first to target the BRD4-NUT fusion protein with ARV-825. The fact that the use of ARV-825 for the treatment of NUT cancer is a novel therapeutic strategy makes it even more exciting.

## 5. Conclusion

In conclusion, our research showed that ARV-825 commenced its action within 12 h on BRD4-NUT 3T3 cells, resulting in inhibited cell proliferation and migration. Moreover, the degradation of BRD4 protein was detectable after ARV-825 continuous administration for 72 h. As a result, the present study indicated that ARV-825 was efficient in inducing BRD4-NUT protein degradation and inhibits the growth of 3T3-BRD4-NUT cells in vitro and vivo. Our research on ectopic expression systems in cell lines has dramatically enhanced our understanding of the molecular alterations that the NUTM1 fusion proteins generated, presented novel perspectives on the creation of new targeting medications, provided theoretical support for personalized treatment, and pointed out promising avenues for targeted therapies facilitate the progress in the treatment of NUT carcinoma. These findings suggested that ARV-825 might be an effective therapeutic approach for treating NUT carcinoma that is fused with BRD4.

## Data Availability

The datasets presented in this study can be found in online repositories. The names of the repository/repositories and accession number(s) can be found as follows: https://www.ncbi.nlm.nih.gov/sra/PRJNA890934.
